# Supplementary material for: Observed Survival Interval: A Supplement to TCGA Pan-Cancer Clinical Data Resource
Source: Cancers (Basel). 2019 Feb 26;11(3):280. doi: 10.3390/cancers11030280 (PMC6468755; doi:10.3390/cancers11030280)
Supplement: Supplementary file 1 [file cancers-11-00280-s001.zip › Supplementary Materials/Supplementary Materials.pdf]

Supplementary Materials

Section I: Data retrieval

The search strategies for clinical data and miRNA isoform sequencing data are as following: (1) project = "TCGA-SKCM"; (2) data category = "Clinical" and "Transcriptome Profiling" for clinical data and miRNA isoform sequencing data, respectively; (3) data type = "Clinical supplement" and "Isoform Expression Quantification" for clinical data, and miRNA isoform sequencing data, respectively; (4) experimental strategy = "miRNA-Seq" for miRNA isoform sequencing data. (5) workflow type = "GCGSC miRNA profiling" for miRNA isoform sequencing data.

Section II: Exclusion criteria

CM patients were excluded by the following criteria: (1) CM patients with no follow-up information or follow-up period less than 60 days; (2) CM patients that had received any preoperative neoadjuvant therapy; (3) CM patients that had prior other non-melanoma malignancy; (4) CM patients that had other non-melanoma malignancy in the follow-up process; (5) CM patients without new tumor in the follow-up but deceased; (6) CM patients that do not have both clinical data and miRNA sequencing data; and (7) CM patients without TCGA sampling time.

Section III: Bioinformatic preprocessing of miRNA sequencing data

MiRNA isoforms only annotated as "mature" were considered to be eligible and those annotated as "precursor", "stemloop", and "unannotated" were discarded. TCGA

sample barcodes were matched by linking “md5” records, which were enclosed in “MANIFEST.txt” and “metadata*.JSON” files. MiRNAs that have at least one count per million expressions in at least 50% CM samples were retained. The miRNA isoform sequencing raw counts were normalized and log2-transformed by R “DESeq2” package.

Section IV: Clinical data preprocessing

The clinical eXtemsible Markup Language files were parsed by R “XML” package into files entitled “clinical_data.csv”, “durgs.csv”, “radiations.csv”, “new_tumor.csv”, and “follow_ups.csv”, respectively. The source R codes for parsing clinical files can be obtained in the supplementary materials of our published paper [15].

Section V: Guided principal component analysis

Guided PCA (gPCA) is an extension of traditional PCA by incorporating batch information into the expression matrix (i.e., batch indicator matrix multiple expression matrix). Instead of performing Singular-value decomposition (SVD) on the expression matrix in traditional PCA, gPCA performing SVD on the batch indicator transformed expression matrix. Large singular values imply that the batch is important for the corresponding principal component. Thus, gPCA guides the SVD to look for batch effects in the data based on the batch indicator matrix. Furthermore, compared to subjective visual inspection of the first and second principal components in traditional PCA, gPCA proposed a statistic (i.e., the ratio of the variance of the first

principal component from gPCA to the variance of the first principal component from traditional PCA) for permutation test. A permutation distribution is created by permuting the batch vector 1000 times so that the test statistic is computed for each permutation. A one-sided P-value is estimated as the proportion of times the observed statistic value was in the extreme tail of the permutation distribution.

Supplementary Figures

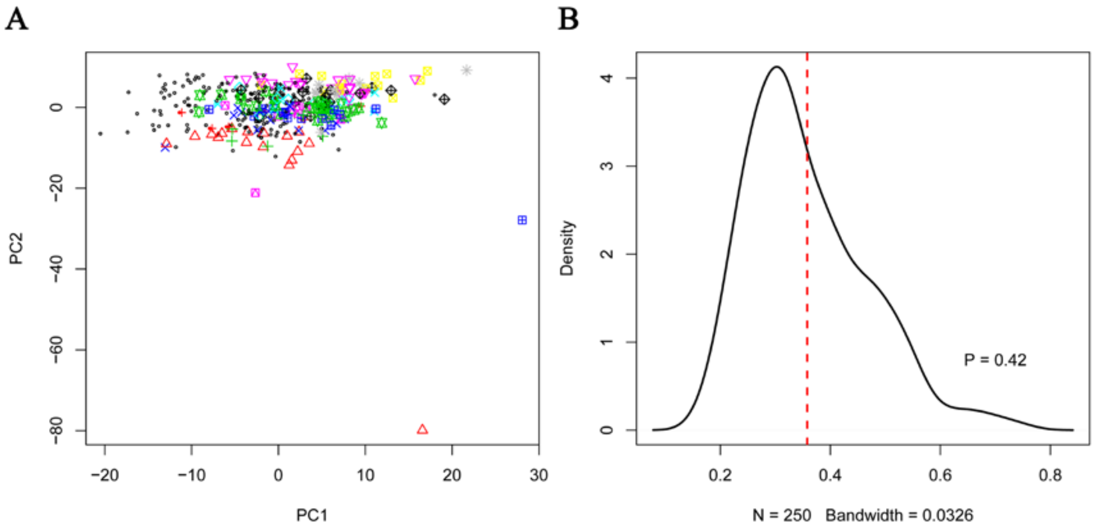

Figure S1. Batch effect evaluation. (**A**) Scatter plot of the first two guided principal components. Different shapes and colors represent different batches. (**B**) Permutation test.

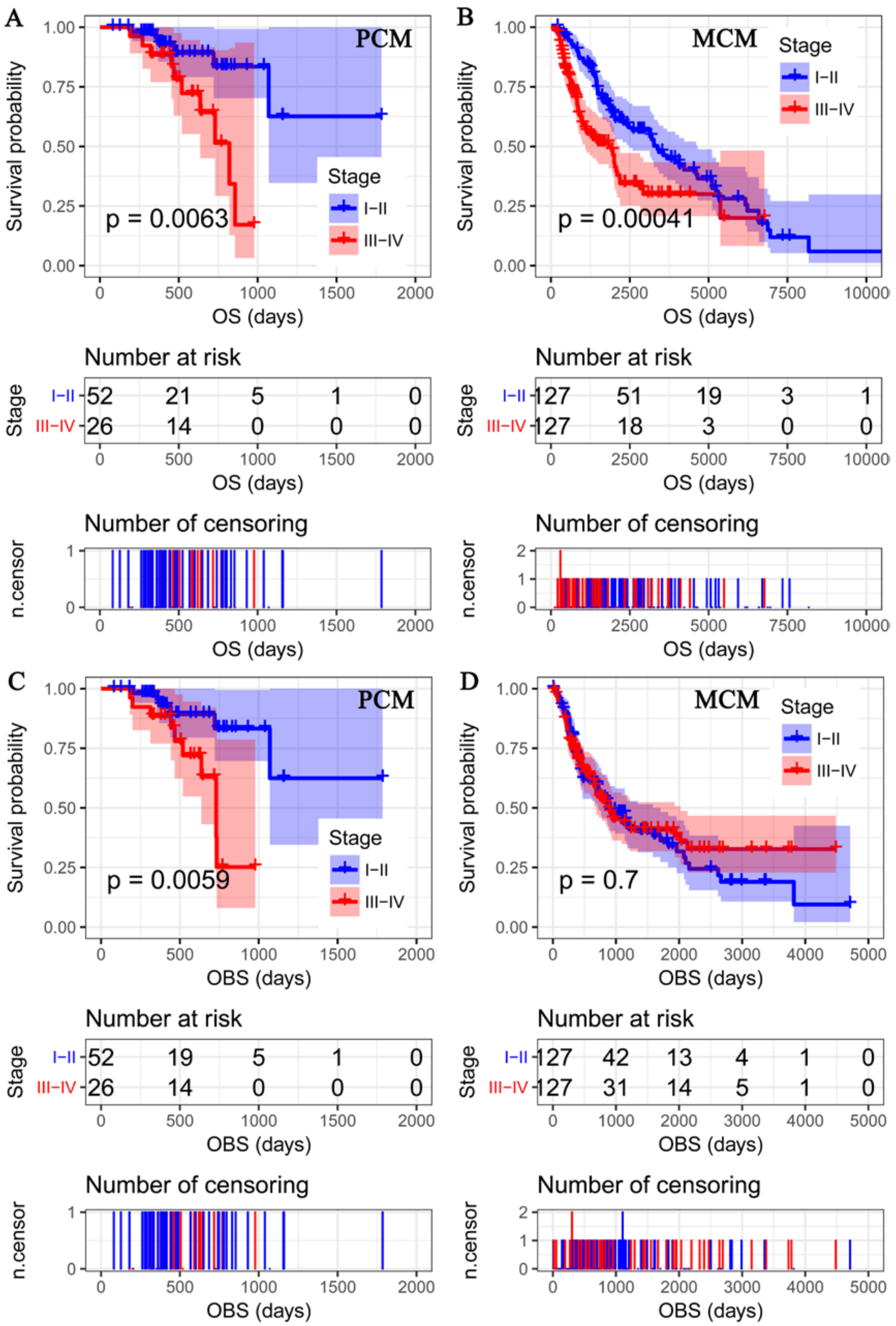

Figure S2. Kaplan-Meier survival analysis of pathological stage. OS and pathological stage in TCGA-PCM cohort (**A**) and TCGA-MCM cohort (**B**). OBS and pathological stage in TCGA-PCM cohort (**C**) and TCGA-MCM cohort (**D**).

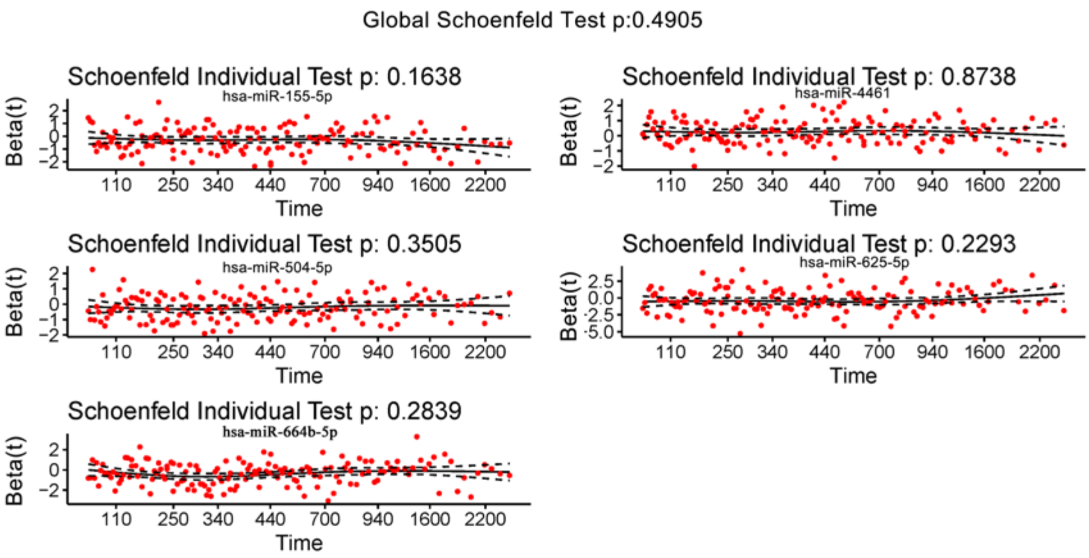

Figure S3. Schoenfeld residuals of hsa-miR-155-5p, hsa-miR-4461, hsa-miR-504-5p, hsa-miR-625-5p, and hsa-miR-664b-5p.

Supplementary Tables

Table S1. Survival analysis of the four OS associated clinicopathological characteristics based on OBS.

	Deaths/Patients (%)	MS (95% CI)	ULog-rank test P
**Age**			
<=50 years	58/113 (51.33)	1061 (854-2022)	
>50 years	115/244 (47.13)	953 (763-1704)	0.506
**Breslow depth**			
<= 2 mm	55/114 (48.25)	1202 (896-2160)	
>2 mm	80/169 (47.33)	777 (703-1070)	0.063
**Pathological stage**			
I-II	81/179 (45.25)	1166 (874-1961)	
III-IV	75/153 (49.02)	854 (691-1929)	0.488
**Ulceration**			
No	57/111 (51.35)	953 (724-2102)	
Yes	58/133 (43.61)	777 (721-1202)	0.542

MS, median survival; CI, confidence interval; U, univariate analysis. Patients with missing values were omitted to display in the table.

Table S2. Univariate analysis of OS associated and OBS associated miRNAs in TCGA-MCM cohort.

	UWald test P	Adjusted P	PH test P	HR (95% CI)
**MCM**				
hsa-let-7c-5p	5.44E-04	0.014	0.89	1.34 (1.14-1.58)
hsa-let-7c-3p	1.74E-03	0.033	0.56	1.22 (1.08-1.39)
hsa-miR-125b-2-3p	1.65E-04	0.006	0.90	1.32 (1.14-1.53)
hsa-miR-142-3p	4.89E-04	0.013	0.41	0.81 (0.71-0.91)
hsa-miR-142-5p	1.52E-04	0.006	0.81	0.78 (0.68-0.89)
hsa-miR-146b-5p	1.84E-03	0.033	0.82	0.73 (0.60-0.89)
hsa-miR-146b-3p	9.36E-05	0.004	0.20	0.66 (0.54-0.81)
hsa-miR-148a-3p	1.02E-03	0.023	0.98	0.78 (0.67-0.91)
hsa-miR-150-3p	6.13E-05	0.003	0.14	0.79 (0.71-0.89)
hsa-miR-150-5p	1.20E-05	<0.001	0.24	0.80 (0.72-0.88)
hsa-miR-151b	1.73E-03	0.033	0.07	0.70 (0.55-0.87)
hsa-miR-155-5p	1.27E-08	<0.0001	0.70	0.68 (0.59-0.78)
hsa-miR-1976	2.69E-06	<0.001	0.99	0.57 (0.45-0.72)
hsa-miR-29b-3p	2.59E-03	0.046	0.59	0.76 (0.64-0.91)
hsa-miR-29c-3p	3.55E-04	0.011	0.44	0.78 (0.68-0.89)
hsa-miR-342-5p	7.04E-07	<0.0001	0.13	0.62 (0.52-0.75)
hsa-miR-342-3p	8.95E-08	<0.0001	0.09	0.62 (0.52-0.74)
hsa-miR-361-3p	6.55E-06	<0.001	0.57	0.55 (0.42-0.71)
hsa-miR-4461	2.03E-04	0.007	0.28	1.29 (1.13-1.47)

hsa-miR-504-5p	5.01E-04	0.013	0.18	0.79 (0.69-0.90)
hsa-miR-505-5p	3.98E-04	0.012	0.32	0.69 (0.56-0.85)
hsa-miR-625-5p	5.70E-08	<0.0001	0.08	0.53 (0.42-0.66)
hsa-miR-625-3p	3.41E-05	0.002	0.18	0.65 (0.53-0.80)
hsa-miR-664b-5p	2.65E-04	0.009	0.21	0.72 (0.61-0.86)
hsa-miR-6720-3p	1.03E-03	0.023	0.92	1.16 (1.06-1.27)
hsa-miR-6842-3p	1.32E-03	0.028	0.74	0.72 (0.59-0.88)
hsa-miR-7702	3.98E-08	<0.0001	0.98	0.74 (0.67-0.83)
**PCM**				
hsa-miR-100-5p	5.17E-04	0.041	0.18	0.82 (0.73-0.92)
hsa-miR-29b-2-5p	1.06E-04	0.015	0.11	0.67 (0.55-0.82)
hsa-miR-29c-5p	6.01E-05	0.011	0.07	0.64 (0.52-0.80)
hsa-miR-3127-5p	2.51E-04	0.024	0.47	1.52 (1.21-1.90)
hsa-miR-3170	7.29E-04	0.046	0.63	1.34 (1.13-1.59)
hsa-miR-3691-5p	5.85E-04	0.041	0.91	1.32 (1.13-1.55)
hsa.mir.4461	8.14E-07	<0.001	0.80	1.40 (1.22-1.60)
hsa.mir.625-5p	2.69E-05	0.008	0.21	0.61 (0.48-0.77)
hsa.mir.7702	2.44E-04	0.024	0.44	0.83 (0.75-0.92)

PH, proportional hazards assumption.

Table S3. Univariate and multivariate analysis of the five cross-validated miRNAs based on OBS.

	^{U}HR (95% CI)	^{U}Wald test P	Adjusted P	^{U}PH test	^{M}HR (95% CI)	^{M}Wald test
miR-142-5p [1]	1.28 (0.68-0.89)	1.523e-04	6.134e-03	0.835	1.08 (0.85-1.38)	0.536
miR-146b-5p [1]	1.37 (0.60-0.89)	1.837e-03	3.343e-02	0.749	1.04 (0.82-1.33)	0.722
miR-150-5p [1]	1.26 (0.72-0.88)	1.197e-05	6.752e-04	0.298	0.96 (0.77-1.18)	0.667
miR-155-5p [2]	1.47 (0.59-0.78)	1.273e-08	7.179e-06	0.743	0.75 (0.61-0.92)	0.005
miR-342-3p [2]	1.61 (0.52-0.74)	8.953e-08	1.262e-05	0.095	0.76 (0.58-0.99)	0.040

PH, proportional hazards assumption; [1], significant in univariate analysis; [2], significant in multivariate analysis.
